# Association Between Funisitis and Childhood Intellectual Development: A Prospective Cohort Study

**DOI:** 10.3389/fneur.2019.00612

**Published:** 2019-06-11

**Authors:** Chengbo Liu, Yan Chen, Dongying Zhao, Jun Zhang, Yongjun Zhang

**Affiliations:** ^1^Department of Neonatology, Xinhua Hospital, Shanghai Jiaotong University School of Medicine, Shanghai, China; ^2^Ministry of Education–Shanghai Key Laboratory of Children's Environmental Health, Xinhua Hospital, Shanghai Jiaotong University School of Medicine, Shanghai, China

**Keywords:** intrauterine infection, fetal inflammatory response syndrome, intelligence quotient, preterm, term

## Abstract

**Background:** Previous studies have suggested that prenatal inflammation could damage the immature brain of preterm infants. In this study, we aimed to investigate whether funisitis could affect childhood neurodevelopment. We hypothesized that childhood neurodevelopment would vary across groups with or without funisitis.

**Material sand Methods:** Using data from the U.S. Collaborative Perinatal Project (1959–1976), 29,725 subjects with available intelligence quotient (IQ) were studied. Detailed placental examinations were conducted according to a standard protocol with quality control procedures. Multivariate logistic regression models were applied to evaluate the relationship between funisitis and IQ at age 4 or 7 years after adjusting for confounders.

**Results:** Early preterm birth children with funisitis had a 3.0-fold (95% confidence interval 1.2, 7.3) risk of low full-scale IQ (<70) at age 4 years, which disappeared until age 7 years. Term birth children with funisitis had 1.9-fold (95% confidence interval 1.2, 3.0) risk of low performance IQ at age 7 years, but they did not have increased risk of low full-scale IQ. No difference in IQ score was found in late preterm birth children.

**Conclusion:** Funisitis may injure the developmental brain of infants, leading to the relative low IQ in childhood at age 4, but the negative effect is only existed in performance IQ at age of 7.

## Introduction

Prenatal infection may result in around 40–70% of preterm deliveries and initiates inflammatory responses that can damage developing brains and other organs (1–3). Several studies on the association between chorioamnionitis and neurological outcomes have been conducted; however, their results are quite heterogeneous (4). Some studies have suggested that chorioamnionitis was associated with preterm births, neurological illnesses, such as white matter lesions and cerebral palsy (5), as well as psychiatric illnesses later in life (6–8). Another study suggested that infants exposed to chorioamnionitis had a high risk of developing abnormal neurological outcomes due to elevation of inflammatory cytokines through the increased permeability of the blood-brain barrier and abnormal myelinization (9). However, Shi et al. drew the opposite conclusions. They found that the evidence for a causal or associative role of chorioamnionitis in cerebral palsy is weak through meta-analysis (10). This discrepancy can be ascribed to the use of different diagnostic criteria for chorioamnionitis exposure and adverse neurological outcomes at various ages, resulting in different inclusion and exclusion criteria among studies (11, 12). Moreover, chorioamnionitis is the maternal response to inflammatory stimuli in the amniotic cavity (13, 14). It is not equated with fetal infection or fetal inflammatory response syndrome (FIRS). Fetal infection is a specific marker for microbial invasion of the amniotic cavity (15).

Funisitis occurs when the inflammatory process involves the umbilical cord. It is accepted that funisitis is a hallmark of FIRS, which is associated with higher rates of neonatal morbidity and multi-organ fetal involvement than chorioamnionitis (13, 16). Recently, studies have attempted to establish, whether, and to what extent, funisitis might negatively affect short- and long-term outcomes of affected infants (17, 18). Most studies focused on primary neurodevelopmental outcomes, including cerebral palsy, in extremely preterm infants; therefore, only a few studies have involved cognitive performance measurements. To further investigate how funisitis may affect childhood neurodevelopment, we used the data from the US Collaborative Perinatal Project (CPP), one of the most comprehensive sources of detailed placental pathology and the largest prospective placental database that includes long-term follow-up of children.

## Materials and Methods

### Study Population

The CPP was a prospective cohort study that recruited pregnant women at 12 university-based academic centers in the United States from 1959 to 1976. The CPP was designed to examine perinatal risk factors for neurologic disorders in children. Women were enrolled at their first prenatal visit, at a mean gestation of 21.3 (SD 8.4) weeks by the last menstrual period, which formed the basis of the gestational age (GA) estimation in the CPP. In-depth demographic, socioeconomic, and behavioral information was collected by in-person interviews by the medical staff taking care of the woman. At the conclusion of the pregnancy, all diagnoses were reviewed and confirmed against pre-specified criteria by a senior study obstetrician at each site.

**Figure 1:**
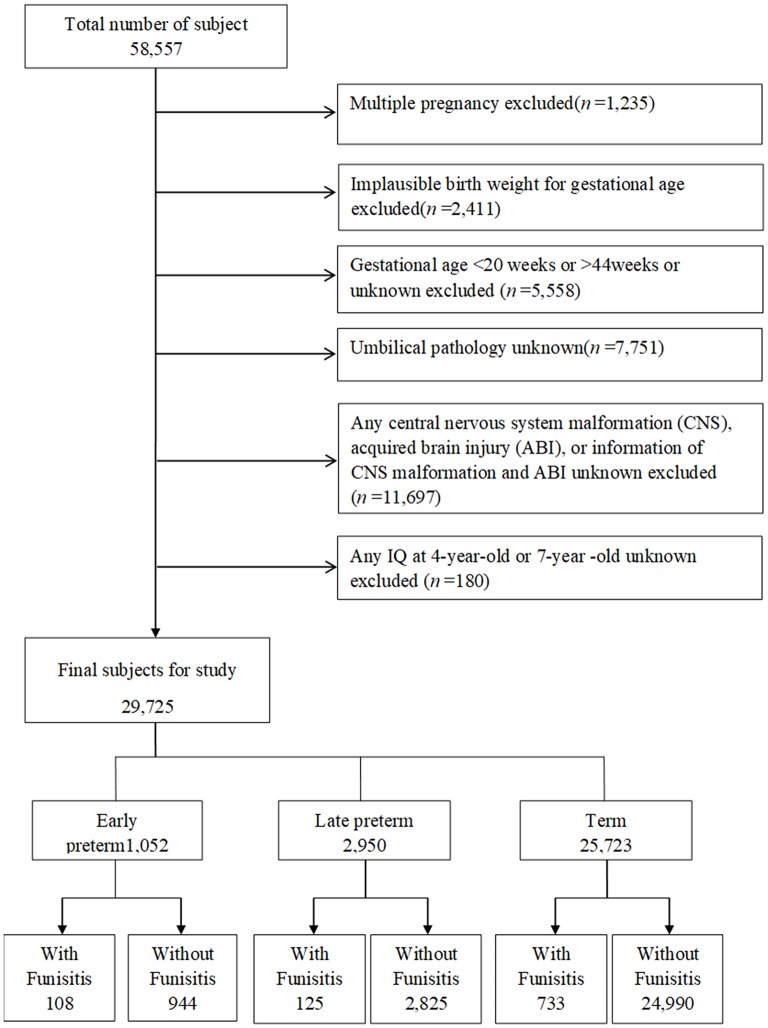
Study flow chart in the selection of study population from the U.S. Collaborative Perinatal Project. Consort diagram of study participants. CNS, central nervous system; ABI, acquired brain injury.

Of the 58,557 pregnancies recruited in CPP, 57,322 singletons (singles births) were identified (Figure 1). The following pregnancies were excluded: singletons with an implausible combination of birth weight for GA (*n* = 2,411) due to likely errors in GA; those with GAs < 20 weeks, older than 44 weeks, or unknown GAs (*n* = 5,558); those with unknown umbilical pathology (*n* = 7,751); and those with any central nervous system (CNS) malformation, acquired brain injury (ABI), CNS malformation, unknown or acquired brain injury (ABI) (*n* = 11,697). After exclusion of subjects with unknown IQ both at age 4 and at age 7 (*n* = 180), this study included 29,725 singletons, including early preterm births (GA at delivery of ≥20 weeks and <34 weeks, *n* = 1,052), late preterm births (GA at delivery of ≥34 weeks and <37 weeks, *n* = 2,950), and term births (GA at delivery of ≥37 weeks and ≤ 44 weeks, *n* = 25,723). Children were followed at age 4 and 7 years with detailed assessments of their neurological, neurosensory, and cognitive development.

### Placental Sample Collection

Placental pathologic assessments described in the CPP were performed by a team of specially trained pathologists and according to a standardized protocol written by Dr. Naeye. Following delivery, umbilical cord gross morphology was examined, and samples were collected for histological examination. Pathologists conducting placental examinations were blinded to the clinical course for 98% of gross and 97% of microscopic examinations. Funisitis was diagnosed as the marked presence of neutrophils in the wall of umbilical vessels with or without Wharton jelly infiltration (19).

### Neurological Development

The CPP conducted the Stanford-Binet intelligence quotient (IQ) test on the study participants at age 4 years. In this study, IQ was also assessed by the Wechsler Intelligence Scale for Children at age 7 years (20).The examinations were highly standardized and followed a strict protocol, with extensive quality control procedures, used by psychologists. full-scale IQ (FSIQ), verbal IQ (VIQ), and performance IQ (PIQ) scores were included in all analyses. The VIQ measured acquired knowledge, comprehension, and verbal reasoning, while the PIQ measured fluid reasoning, spatial sequencing, attention to detail, and visual-motor integration. The FSIQ score was based on a combination of the VIQ and PIQ scores. The IQ score <70 was defined as intellectual disability, and IQ score not < 90 was considered normal (21).

### Confounders

Perinatal factors that may affect the relationship between funisitis and child IQ scores were chosen as potential confounders according to a previous literature (21). Maternal characteristics included maternal age at delivery (<20, 20–24, 25–29, 30–34, or ≥35 years), race (white, black, or other), parity (0, 1, or ≥ 2), marital status at pregnancy (no/yes), smoking during pregnancy (no/yes), educational level (<9, 10–12, or >12 years), maternal BMI (<18.5, 18.5–25, 25–30, or ≥ 30 kg/m^2^), and diabetes (no/yes). The socioeconomic status index was a combined score of maternal education, occupation, and family income and was further classified into five categories (22). Blood pressure was recorded at study entry, each pre-pregnancy visit, during labor and delivery, and postpartum. Preeclampsia/eclampsia was defined as gestational hypertension plus any of the following documented symptoms: gestational proteinuria, oliguria, pulmonary edema, or convulsions from 25 weeks of gestation to 5 weeks postpartum, and was based on the actual blood pressure values recorded in the data files, rather than on diagnostic summaries completed at that time. Detailed descriptions have been provided elsewhere (23). The diagnosis of diabetes was based on the medical records in the CPP.

### Data Sharing Statement and Ethical Approval

Since the data of the CPP is publicly available, ethics approval is exempted from review by our Institutional Review Board.

### Statistical Analysis

Using the chi-squared test, we first examined the relationship between maternal baseline characteristics and perinatal outcomes, and child IQ (< 70 vs. ≥90). All confounders were selected a priori and evaluated for associations with funisitis and the intellectual function measures, respectively. We then conducted multivariate logistic regression analyses to evaluate the exposure-response relationship between funisitis and IQ and adjusted for confounders (i.e., maternal race, maternal age, parity, marital status, social economic status, educational level, smoking during pregnancy, maternal prepregnant BMI, neonatal sex, and gestational age).Additionally, similar multivariate logistic regression analyses were performed when IQ was classified by 10th, 5th, 3rd, and 1st percentiles to confirm the veritable association between funisitis and IQ, rather than using the standard low IQ definition (<70) by chance.

## Results

The study population selection process is illustrated in Figure 1. A total of 29,725 subjects with available IQ scores were included in the analysis. Mothers of children with IQ≥90 were more likely to be more than 20 years old, white, married, and multiparous and have higher educational level, higher social economic status, and normal maternal BMI than those of children with IQ <70 (Table 1). In addition, the incidence of IQ <70 was higher in early preterm birth than in late preterm birth or term birth.

**Table 1:** Maternal characteristics and perinatal outcomes by IQ.

	**IQ at 4 years**				**IQ at 7 years**			
**Characteristics**	**IQ ≥90**	**IQ <70**	**χ^2^**	**P**	**IQ ≥90**	**IQ <70**	**χ^2^**	**P**
N(%)	18,656 (95.4)	904 (4.6)			20,447 (96.3)	784 (3.7)		
Maternal age (year)			90.1	<0.0001			28.5	<0.0001
<20	3,645 (19.5)	290 (32.1)			4,076 (19.9)	212 (27.0)		
20–24	6,634 (35.6)	295 (32.6)			7,528 (36.8)	242 (30.9)		
25–29	4,266 (22.9)	146 (16.2)			4,569 (22.4)	159 (20.3)		
30–34	2,462 (13.2)	103 (11.4)			2,555 (12.5)	107 (13.7)		
≥ 35	1,649 (8.8)	70 (7.7)			1,719 (8.4)	64 (8.2)		
Race			499.9	<0.0001			629.7	<0.0001
White	10,763 (57.6)	180 (19.9)			12,527 (61.3)	134 (17.1)		
Black	7,305 (39.2)	666 (73.7)			7,253 (35.5)	617 (78.7)		
Other	588 (3.2)	58 (6.4)			667 (3.3)	33 (4.2)		
Parity			32.7	<0.0001			64.2	<0.0001
0	5,260 (28.3)	311 (34.6)			6,200 (30.4)	185 (23.7)		
1	4,412 (23.7)	146 (16.2)			4,908 (24.1)	127 (16.3)		
≥ 2	8,944 (48.0)	443 (49.2)			9,296 (45.6)	468 (60.0)		
Social economic status			584.7	<0.0001			746.0	<0.0001
1 (Lowest)	923 (5.0)	160 (18.4)			926 (4.6)	150 (19.9)		
2	4,555 (24.8)	382 (43.7)			4,623 (23.0)	363 (48.1)		
3	5,832 (31.8)	256 (29.4)			6,254 (31.1)	188 (24.9)		
4	4,505 (24.5)	60 (6.9)			5,161 (25.7)	45 (6.0)		
5 (Highest)	2,552 (13.9)	14 (1.6)			3,146 (15.6)	8 (1.1)		
Maternal education levels (year)			350.7	<0.0001			500.3	<0.0001
Less than high school (≤9)	4,229 (22.7)	436 (48.2)			4,409 (21.6)	424 (54.1)		
High school (10–12)	11,748 (62.9)	446 (49.6)			12,655 (61.9)	352 (44.9)		
College and above (> 12)	2,679 (14.4)	22 (2.4)			3,383 (16.6)	8 (1.0)		
Married	15,198 (81.5)	598 (66.2)	130.1	<0.0001	16,877 (82.5)	504 (64.3)	169.5	<0.0001
Smoking during pregnancy	8,958 (48.3)	380 (42.4)	11.9	0.001	9,735 (47.9)	329 (42.4)	9.0	0.003
Maternal BMI (kg/m2)			3.7	0.295			27.8	<0.0001
<18.5	2,594 (14.1)	107 (12.2)			3,071 (15.2)	89 (11.9)		
18.5–25	11,863 (64.4)	561 (64.2)			13,038 (64.6)	455 (60.8)		
25–30	2,637 (14.3)	139 (15.9)			2,728 (13.5)	126 (16.8)		
≥ 30	1,331 (7.2)	67 (7.7)			1,343 (6.7)	78 (10.4)		
Preterm delivary			76.5	<0.0001			153.2	<0.0001
Early preterm	458 (2.4)	54 (6.0)			18,192 (89)	598 (76.3)		
Late preterm	1,617 (8.7)	127 (14.0)			1,754 (8.6)	120 (15.3)		
Term	16,581 (88.9)	723 (80.0)			501 (2.5)	66 (8.4)		
Preeclampsia	425 (2.3)	32 (3.6)	6.1	0.014	463 (2.3)	30 (3.9)	8.2	0.004
Chronic hypertension	3,727 (20.0)	179 (19.9)	0.0	0.931	4,083 (20.0)	172 (22.1)	1.9	0.166
Gestational diabetes	438 (2.4)	11 (1.2)	4.9	0.027	483 (2.4)	11 (1.4)	3.0	0.081
Preexisting diabetes	290 (1.6)	9 (1.0)	1.8	0.181	324 (1.6)	7 (0.9)	2.3	0.126

*IQ, intelligence quotient; Early preterm, Gestational age at birth <34 weeks*;

*Late preterm, Gestational age at birth ≥34 and <37 weeks*;

*Term, Gestational age at birth ≥37 and ≤ 44 weeks*.

In our dataset, there were 966 children with funisitis among the 29,725 subjects, with an overall prevalence of 3.25%. However, using the intellectual outcomes measured as categorical variables in adjusted logistic regression models, in early preterm birth children, compared with children without funisitis, those with funisitis have a 3.0-fold (95% confidence interval (CI) 1.2, 7.3) risk of low IQ (<70) at age 4 years, but this risk disappears as children reached 7 years of age. In term birth children, those with funisitis have moderate high risk [1.9-fold (95% CI 1.2, 3.0)] of low PIQ at 7 years of age, but no risk in FSIQ. In late preterm birth children, no difference in IQ was found, neither at age 4 nor at age 7 years (Table 2). Since only one definition for low IQ (IQ score <70) is not very convincing, we evaluated the associations between funisitis and diverse definitions of low IQ, in order to confirm the veritable associations. Appendix Table 1 shows the IQ value distributions at the 10th, 5th, 3rd, and 1st percentiles. When IQ was lower than the 10th, 5th, 3rd, and 1st percentiles as outcome, multivariate logistic regression analysis showed similar results (Appendix Table 2).

**Table 2:** Risk of low IQ (score <70)^#^ at age 7 years in children with funisitis.

		**Without funisitis N (%) ref**	**With funisitis N (%)**	**Crude OR (95% CI)**	Adjusted OR^*^ (95% CI)
IQ at 4 Years	Early Preterm	45 (5.8)	9 (10.5)	1.5 (0.7, 3.3)	***3.0 (1.2, 7.3)***
	Late preterm	124 (5.1)	3 (3.2)	0.6 (0.2, 1.9)	0.6 (0.1, 2.4)
	Term	706 (3.3)	17 (2.8)	0.8 (0.5, 1.3)	1.1 (0.6, 1.9)
FSIQ at 7 Years	Early Preterm	59 (6.3)	7 (6.7)	1.0 (0.4, 2.2)	1.4 (0.6, 3.6)
	Late preterm	114 (4.1)	6 (4.9)	1.1 (0.5, 2.6)	1.3 (0.5, 3.2)
	Term	578 (2.3)	20 (2.8)	1.1 (0.7, 1.7)	1.5 (0.9, 2.4)
VIQ at 7 Years	Early Preterm	68 (7.3)	7 (6.7)	0.9 (0.4, 2.0)	1.3 (0.5, 3.2)
	Late preterm	128 (4.6)	7 (5.7)	1.2 (0.5, 2.6)	1.3 (0.5, 3.1)
	Term	628 (2.6)	21 (2.9)	1.0 (0.7, 1.6)	1.2 (0.8, 2.0)
PIQ at 7 Years	Early Preterm	59 (6.4)	8 (7.8)	1.1 (0.5, 2.4)	1.6 (0.7, 3.9)
	Late preterm	121 (4.4)	5 (4.1)	0.9 (0.3, 2.2)	1.0 (0.4, 2.6)
	Term	617 (2.5)	25 (3.5)	1.3 (0.9, 2.0)	***1.9 (1.2, 3.0)***

*CI, confidence interval; OR, odds ratio; FSIQ, full-scale intelligence quotient; IQ, intelligence quotient; PIQ, performance intelligence quotient; VIQ, verbal intelligence quotient*.

# *Outcome: IQ < 70 and IQ ≥90*.

* *Adjusted for maternal race, maternal age, parity, marital status, social economic status, educational level, smoking during pregnancy (any), maternal prepregnant BMI, neonatal sex, and gestational age. Early preterm: Gestational age at birth <34 weeks. Late preterm: Gestational age at birth ≥34 and <37 weeks. Term: Gestational age at birth ≥37 and ≤ 44 weeks*.

*Bold and italic values indicates statistically significant*.

## Discussion

This study analyzed the associations between funisitis and long-term childhood neurodevelopment. Early preterm birth children with funisitis had a 3.0-fold risk of low IQ (<70) at age 4 years, but the risk decreased significantly as the children grew. In term birth children, those with funisitis had 1.9-fold risk of low PIQ at age 7 years; however, they did not have a significant risk of low FSIQ. No difference in IQ score was found in late preterm birth children.

Chorioamnionitis, a common etiology of preterm birth infants, has often been cited as a risk factor for the development of cerebral palsy and brain injury, utilizing animal models and epidemiological surveys (24–26). Any evidence of mural inflammation in the umbilical cord, as an independent risk factor for encephalopathy (27), which is more harmful than chorioamnionitis alone, is consistent with FIRS and considered of fetal origin directly (28). However, most studies are focused on primary neurodevelopmental disorders and short-term outcomes. Jessop et al. (16) found that funisitis in live birth deliveries at or near term is associated with adverse clinical outcomes, but they lacked long-term follow-up surveys. In a study of 225 early preterm infants, Rovira et al. (29) reported that funisitis may entail a higher risk of moderate to severe neurological disability at age 2 years, but they did not conduct further analysis due to interrupted follow-ups. A small sample-sized research study on genomic biomarkers in umbilical cord tissue, identified that funisitis was associated with altered gene expression and neurocognitive function at 10 years of age (30). Our study attempted to analyze in depth the effects of funisitis on long-term neurodevelopment of children using CPP dataset, including almost 30,000 children with IQ available from birth through age 7 years. We found that early preterm babies may have a high risk of long-term adverse effects on childhood intellectual development at age 4 years, probably because acute funisitis in preterm placenta was associated with severe FIRS (17) and FIRS may be responsible for neonatal adverse neurological outcomes (31, 32), such as cerebral palsy, CNS malformations, and behavior abnormalities. As children reached to 7 years of age, general intelligence seemed to be mostly abrogated, presumably due to postnatal intentional training (33). Another possible explanation maybe the variations between the Standford-Binet Intelligence Test and the Wechsler Intelligence Scale for Children based on different theories (34).

Interestingly, the high risk of low PIQ was confirmed in children with funisitis in this study. VIQ scores are thought to reflect language processing, including acquired knowledge, comprehension, and verbal reasoning, and is likely involved in general intelligence, reading skills, and verbal ability. PIQ scores are used to assess planning, logical thinking, spatial analysis, and visual-motor integration. Infants with neonatal nerve injuries are often associated with impairments in visual attention and visuospatial processing related to PIQ scores, but neither FSIQ nor VIQ scores (35, 36). Visible motor-related problems are roughly consistent with the location of injuries near the head of the caudate nucleus (37). Children with attention-deficit hyperactivity disorder are associated with lower PIQ scores (38). They have activation suppression of the caudate nucleus and prefrontal regions of the brain, including the right inferior regions. Thus, we speculate that the caudate nucleus may be one of the vulnerable sites to FIRS in term birth, and damage of the caudate nucleus may result in lower PIQ scores. Moreover, children's cognitive abilities undergo considerable improvements from infant to adolescence, and VIQ and PIQ scores may have different developmental trajectories. A combination of genes and common environmental factors play important roles in neurological development over time (39). In a prospective study of more than 100 twins and their older siblings, the authors reported that heritability could explain 34% of their FSIQ, 37% of their VIQ, and 64% of their PIQ scores (40). Improvements in VIQ scores were more likely due to common environmental factors than PIQ, including maternal education, neighborhood characteristics, and social economic status (40).

In our study, no significant difference was found in the groups of late preterm and term children with funisitis at the age of 4 years-old. The increasing grade of funisitis with the aggravating FIRS was reported to be related to an earlier GA at delivery and severer brain injury (13, 41). Compared to early preterm infants, late preterm and term infants may not within the window in which the developing brain was vulnerable to severe inflammatory injury. Moreover, FSIQ is affected by a variety of factors, including environmental and genetic factors (39) and mild brain injury in late preterm and term infants may not strong enough to counteract the influence of environmental and genetic factors. Therefore, it is plausible that we cannot find positive correlations between funisitis and FSIQ in late preterm and term infants. When we used the Wechsler Intelligence Scale and FSIQ was divided into PIQ and VIQ, the effect of funisitis on PIQ, which is less associated with heritability, was disclosed. In the CPP, however, since the IQ score at age 4 did not include PIQ and VIQ, we cannot tell whether difference of PIQ was existed in children at age 4. The reason why difference of PIQ at age 7 was existed only in term birth, the mechanism is not clear, probably related to the small number of subjects with funisitis assigned into preterm subgroups. Further studies on the mechanism are warranted.

Although the CPP was conducted 60 years ago, it has the largest collection of maternal-birth data, including maternal characteristics, medication, and childhood growth and developmental health measures. As the largest prospective birth cohort study in the United States, the CPP was carefully conducted with a high long-term follow-up rate (79% by age 7 years) (42). Lower cognitive scores in children have been found to be generally stable beginning at age 4 (43), but with greater intra- and inter- individual variability. Additional behavioral problems such as emotional symptoms, hyperactivity/inattention, and peer problems are also associated with cognitive impairments, which become apparent by age 5 years (44). Thus, the neurodevelopment assessment of children at age 4 and 7 years was stable and credible. In this study, assessments were highly standardized based on strict protocols with extensive quality control procedures in almost 30,000 children from birth through age 7 years. These strengths made the CPP dataset particularly suitable for studying the association between funisitis and child intellectual development. A major challenge to the neurodevelopment outcomes due to funisitis was how to deal with the role of confounders that also influence cognitive development. Our study included genetic and environmental confounders according to previous literature. To balance the differences in neurodevelopmental maturity, we divided our study population into three subgroups: early preterm birth group, late preterm group, and term birth group. To the best of our knowledge, the present study is the first to report the longitudinal effect of funisitis on children intellectual function at age 7 years.

This study has some limitations. An inherent study limitation is that we do not have any information on the severity of the funisitis. A study of the histologic characteristics of funisitis associated with neurodevelopment demonstrated that a severe fetal inflammatory response was associated with higher mortality and lower IQ scores (28). In this study, the severity of funisitis was classified by increasing stages: stage 1, phlebitis; stage 2, arteritis with or without phlebitis; and stage 3, subacute necrotizing funisitis. However, reports of the relationship between inflammation severity and adverse neurological outcomes have not been consistent. Huetz et al. (45) reported no significant association between severe placental inflammatory lesions and cerebral palsy. Additionally, when subjects with funisitis were categorized according to GA at delivery, each subgroup had a small number of participants, which may influence the results of statistical analysis. Finally, on account that different IQ tests were administered, the Stanford-Binet for children at age 4 and the WISC for children at age 7, differences in performance across the 2 ages could be related to differences in the IQ batteries (34). Thus the IQ scores can not be directly comparable.

## Conclusion

Funisitis may not have significant long-term effects on general intellectual development, even with early preterm birth. Early preterm birth children with funisitis had a significant high risk of low IQ (<70) at age 4 years, but the risk decreased significantly as the children grew up. In term birth children, prenatal inflammation may have certain effects on PIQ at age 7, but not on FSIQ. Specific training for term birth infants with intrauterine infection in spatial sequencing and problem-solving skills may be beneficial to improve their PIQ scores.

## Ethics Statement

Since the data of the Collaborative Perinatal Project is publicly available, ethics approval is exempt from review by our Institutional Review Board.

## Author Contributions

YZ and JZ designed the study. YC and DZ performed the statistical analyses. CL checked the statistics and wrote the first draft. All authors critically revised the manuscript and approved the final version. In addition, each author certifies that he or she has participated sufficiently in the work to believe in its overall validity and to take public responsibility for appropriate portions of the content.

### Conflict of Interest Statement

The authors declare that the research was conducted in the absence of any commercial or financial relationships that could be construed as a potential conflict of interest.
