# Supplementary material for: Association Between Funisitis and Childhood Intellectual Development: A Prospective Cohort Study
Source: Front Neurol. 2019 Jun 11;10:612. doi: 10.3389/fneur.2019.00612 (PMC6584799; doi:10.3389/fneur.2019.00612)
Supplement: Supplementary file 1 [file Table_1.DOCX]

**Appendix Table1:** IQ value distribution at 10^th^ percentile, 5^th^ percentile, 3^th^ percentile and 1^th^ percentile

|  | FSIQ at 4 years | FSIQ at 7 years | VIQ at 7 Years | PIQ at 7 Years |
| --- | --- | --- | --- | --- |
| 10^th^ percentile | 78 | 79 | 79 | 79 |
| 5^th^ percentile | 72 | 73 | 74 | 75 |
| 3^th^ percentile | 68 | 70 | 70 | 72 |
| 1^th^ percentile | 60 | 62 | 63 | 65 |

Abbreviations: IQ, intelligence quotient; FSIQ, full-scale intelligence quotient; VIQ, verbal intelligence quotient; PIQ, performance intelligence quotient.
