# Supplementary material for: Association Between Funisitis and Childhood Intellectual Development: A Prospective Cohort Study
Source: Front Neurol. 2019 Jun 11;10:612. doi: 10.3389/fneur.2019.00612 (PMC6584799; doi:10.3389/fneur.2019.00612)
Supplement: Supplementary file 2 [file Table_2.DOCX]

**Appendix Table2:** Odds ratios for low IQ by percentile.

|  |  | Without funisitis | With funisitis | Crude OR | Adjusted OR* |
| --- | --- | --- | --- | --- | --- |
|  |  | N(%) ref | N(%) | (95%CI) | (95%CI) |
| **IQ at 4 Years** |  |  |  |  |  |
| <78 (10^th^ percentile) | Early Preterm | 137 (16.7) | 15 (16.3) | 1.0 (0.5, 1.7) | 1.3 (0.7, 2.4) |
|  | Late preterm | 301 (11.7) | 6 (5.4) | 0.4 (0.2, 1.0) | 0.4 (0.2, 1.1) |
|  | Term | 1671 (7.1) | 36 (5.2) | 0.7 (0.5, 1.0) | 0.9 (0.6, 1.3) |
| <72 (5^th^ percentile) | Early Preterm | 61 (7.0) | 9 (9.0) | 1.3 (0.6, 2.8) | 2.2 (1.0, 4.8) |
|  | Late preterm | 152 (5.6) | 4 (3.4) | 0.6 (0.2, 1.6) | 0.6 (0.2, 1.9) |
|  | Term | 831 (3.4) | 19 (2.7) | 0.8 (0.5, 1.2) | 0.9 (0.6, 1.5) |
| <68 (3^th^ percentile) | Early Preterm | 33 (3.7) | 9 (8.6) | ***2.5 (1.1, 5.3)*** | ***4.0 (1.7, 9.3)*** |
|  | Late preterm | 91 (3.3) | 2 (1.7) | 0.5 (0.1, 2.1) | 0.7 (0.2, 2.9) |
|  | Term | 527 (2.2) | 14 (1.9) | 0.9 (0.5, 1.5) | 1.0 (0.6, 1.8) |
| <60 (1^th^ percentile) | Early Preterm | 12 (1.3) | 3 (2.8) | 2.2 (0.6, 7.9) | ***6.1 (1.3, 28.6)*** |
|  | Late preterm | 31 (1.1) | 1 (0.8) | 0.7 (0.1, 5.4) | 1.0 (0.1, 7.7) |
|  | Term | 154 (0.6) | 7 (1.0) | 1.6 (0.7, 3.3) | 1.9 (0.9, 4.1) |
| **FSIQ at 7 Years** |  |  |  |  |  |
| <79 (10^th^ percentile) | Early Preterm | 181 (19.4) | 23 (22.1) | 1.2 (0.7, 1.9) | 1.6 (0.9, 2.7) |
|  | Late preterm | 382 (13.7) | 16 (13.0) | 0.9 (0.6, 1.6) | 1.0 (0.6, 1.8) |
|  | Term | 2189 (8.9) | 63 (8.7) | 1.0 (0.8, 1.3) | 1.3 (1.0, 1.7) |
| <73 (5^th^ percentile) | Early Preterm | 99 (10.6) | 13 (12.5) | 1.2 (0.6, 2.2) | 1.6 (0.8, 3.1) |
|  | Late preterm | 185 (6.6) | 7 (5.7) | 0.9 (0.4, 1.9) | 1.0 (0.4, 2.2) |
|  | Term | 981 (4.0) | 36 (5.0) | 1.3 (0.9, 1.8) | ***1.6 (1.1, 2.4)*** |
| <70 (3^th^ percentile) | Early Preterm | 59 (6.3) | 7 (6.7) | 1.1 (0.5, 2.4) | 1.5 (0.6, 3.5) |
|  | Late preterm | 114 (4.1) | 6 (4.9) | 1.2 (0.5, 2.8) | 1.5 (0.6, 3.5) |
|  | Term | 578 (2.3) | 20 (2.8) | 1.2 (0.8, 1.9) | 1.5 (0.9, 2.4) |
| <62 (1^th^ percentile) | Early Preterm | 17 (1.8) | 1 (1.0) | 0.5 (0.1, 4.0) | 0.8 (0.1, 6.1) |
|  | Late preterm | 38 (1.4) | 2 (1.6) | 1.2 (0.3, 5.0) | 1.3 (0.3, 5.8) |
|  | Term | 185 (0.8) | 9 (1.2) | 1.7 (0.8, 3.3) | 1.9 (0.9, 4.0) |
| **VIQ at 7 Years** |  |  |  |  |  |
| <79 (10^th^ percentile) | Early Preterm | 183 (19.7) | 22 (21.2) | 1.1 (0.7, 1.8) | 1.5 (0.9, 2.7) |
|  | Late preterm | 389 (14.0) | 18 (14.6) | 1.1 (0.6, 1.8) | 1.1 (0.6, 2.0) |
|  | Term | 2225 (9.1) | 55 (7.6) | 0.8 (0.6, 1.1) | 1.1 (0.8, 1.4) |
| <74 (5^th^ percentile) | Early Preterm | 106 (11.4) | 10 (9.6) | 0.8 (0.4, 1.6) | 1.2 (0.6, 2.5) |
|  | Late preterm | 198 (7.1) | 10 (8.1) | 1.2 (0.6, 2.2) | 1.2 (0.6, 2.5) |
|  | Term | 1102 (4.5) | 28 (3.9) | 0.9 (0.6, 1.3) | 1.0 (0.7, 1.6) |
| <70 (3^th^ percentile) | Early Preterm | 68 (7.3) | 7 (6.7) | 0.9 (0.4, 2.0) | 1.4 (0.6, 3.2) |
|  | Late preterm | 128 (4.6) | 7 (5.7) | 1.2 (0.6, 2.7) | 1.4 (0.6, 3.2) |
|  | Term | 628 (2.6) | 21 (2.9) | 1.1 (0.7, 1.8) | 1.5 (0.9, 2.4) |
| <63 (1^th^ percentile) | Early Preterm | 26 (2.8) | 2 (1.9) | 0.7 (0.2, 2.9) | 1.3 (0.3, 6.1) |
|  | Late preterm | 41 (1.5) | 3 (2.4) | 1.7 (0.5, 5.5) | 2.1 (0.6, 7.2) |
|  | Term | 201 (0.8) | 6 (0.8) | 1.0 (0.4, 2.3) | 1.3 (0.5, 3.2) |
| **PIQ at 7 Years** |  |  |  |  |  |
| <79 (10^th^ percentile) | Early Preterm | 150 (16.2) | 14 (13.6) | 0.8 (0.5, 1.5) | 0.9 (0.5, 1.7) |
|  | Late preterm | 287 (10.4) | 11 (8.9) | 0.9 (0.5, 1.6) | 0.9 (0.5, 1.8) |
|  | Term | 1700 (6.9) | 52 (7.2) | 1.0 (0.8, 1.4) | 1.3 (0.9, 1.8) |
| <75 (5^th^ percentile) | Early Preterm | 74 (8.0) | 11 (10.7) | 1.4 (0.7, 2.7) | 1.7 (0.8, 3.5) |
|  | Late preterm | 160 (5.8) | 9 (7.3) | 1.3 (0.6, 2.6) | 1.5 (0.7, 3.0) |
|  | Term | 913 (3.7) | 31 (4.3) | 1.2 (0.8, 1.7) | ***1.5 (1.1, 2.2)*** |
| <72 (3^th^ percentile) | Early Preterm | 59 (6.4) | 8 (7.8) | 1.2 (0.6, 2.7) | 1.7 (0.7, 3.9) |
|  | Late preterm | 121 (4.4) | 5 (4.1) | 0.9 (0.4, 2.3) | 1.0 (0.4, 2.6) |
|  | Term | 617 (2.5) | 25 (3.5) | 1.4 (0.9, 2.1) | ***1.8 (1.2, 2.8)*** |
| <65 (1^th^ percentile) | Early Preterm | 22 (2.4) | 1 (1.0) | 0.4 (0.1, 3.0) | 0.6 (0.1, 4.7) |
|  | Late preterm | 38 (1.4) | 0 (0) | / | / |
|  | Term | 209 (0.9) | 14 (1.9) | ***2.3 (1.3, 4.0)*** | ***3.0 (1.6, 5.4)*** |

Abbreviations: CI, confidence interval; OR, odds ratio; IQ, intelligence quotient; FSIQ, full-scale intelligence quotient; VIQ, verbal intelligence quotient; PIQ, performance intelligence quotient.

* Adjusted for maternal race, maternal age, parity, marital status, social economic status (SES), education levels, smoking during pregnancy (any), maternal prepregnant BMI, neonatal gender and gestational age.

Early preterm: Gestational age at birth < 34 weeks.

Late preterm: Gestational age at birth ≥34 weeks and < 37 weeks.

Term: Gestational age at birth ≥37 weeks and≤ 44 weeks.

.
